# Recurrent, life-threatening PE in the setting of popliteal vein aneurysm in pregnancy: a case report

**DOI:** 10.1186/s12959-023-00495-2

**Published:** 2023-05-05

**Authors:** S. Ainslie McBride, Luke A. Rannelli, Paul M. Cantle

**Affiliations:** 1 Department of Medicine, University of Calgary, Richmond Road Diagnostic and Treatment Centre, 1820 Richmond Rd SW, Calgary, AB T2T 5C7 Canada; 2 Department of Medicine, University of Calgary, Rockyview General Hospital, 7007 14 St SW, Calgary, AB T2V 1P9 Canada; 3 Department of Surgery, University of Calgary, Peter Lougheed Centre 5Th East Wing, 5940 - 3500 26th Ave NE, Calgary, AB T1Y 6J4 Canada

**Keywords:** Popliteal vein aneurysms, Venous thromboembolic event, Pulmonary embolism, Pregnancy, Anticoagulation

## Abstract

**Background:**

Popliteal vein aneurysms (PVA) are a rare clinical entity with unknown etiology that pose a significant risk for venous thromboembolic events (VTE). The current literature supports anticoagulation and operative management. There are few case reports of PVA in pregnancy. We present a unique case of a pregnant patient with recurrent pulmonary embolism (PE) in the setting of PVA with intra-aneurysmal thrombosis who ultimately underwent surgical excision.

**Case presentation:**

A previously healthy 34-year-old G2P1 at 30 weeks gestation presented to the emergency department with shortness of breath and chest pain. She was diagnosed with PE and subsequently required intensive care unit (ICU) admission and thrombolysis for a massive PE. While on a therapeutic dose of tinzaparin she had recurrence of PE in the post-partum period. She was treated with supratherapeutic tinzaparin and subsequently transitioned to warfarin. She was found to have a PVA and ultimately underwent successful PVA ligation. She remains on anticoagulation for secondary prevention of VTE.

**Conclusions:**

PVA are a rare but potentially fatal source of VTE. Patients most commonly present with symptoms of PE. The risk of VTE is elevated in the pro-thrombotic states of pregnancy and the post-partum period due to both physiologic and anatomical changes. The recommended management of PVA with PE is anticoagulation and surgical resection of the aneurysm, however this can be complicated in the setting of pregnancy. We demonstrated that pregnant patients with PVA can be temporized with medical management to avoid surgical intervention during pregnancy, but require close symptom monitoring and serial imaging to reassess the PVA, with high index of suspicion for recurrent VTE. Ultimately, patients with PVA and PE should undergo surgical resection to reduce the risk of recurrence and long-term complications. The ideal duration of post-operative anticoagulation remains unclear, and should likely be decided on based on risks, benefits, values, and shared decision making with the patient and their care provider.

## Background

Popliteal vein aneurysms (PVA), defined by an isolated, persistent venous dilation of greater than twice the size of the native vein, are a rare clinical entity with reported incidence of 0.2–0.5%, and are more common in women [1–3]. The etiology is unknown, but possible causes include trauma, inflammation, or congenital – including vascular wall weakness due to elastin insufficiency and malformations [2].

PVA increase the risk of venous thromboembolic events (VTE), and despite the location, rarely manifest as pain and/or leg swelling. Instead, 24–75% of patients with PVA present with symptoms confirmed to be secondary to pulmonary embolism (PE), and 43–80% of patients have been reported to have recurrent PE despite therapeutic doses of anticoagulation [2–6].

Few cases of PVA have been reported in the setting of pregnancy. The risk of thrombotic events in patients with pre-existing risk factors such as vascular anomalies is further increased in the prothrombotic state of pregnancy. We present a unique case of PVA in a previously healthy pregnant patient with recurrent PE who ultimately underwent surgical excision.

## Case presentation

A previously healthy 34-year-old G2P1 at 30 weeks gestation presented to the emergency department (ED) with shortness of breath and chest pain. She was diagnosed with large burden pulmonary embolism (PE) on Ventilation/Perfusion Scan (V/Q scan). Her first pregnancy had been uncomplicated, with no previous history of venous thromboembolic events (VTE). She had received the mRNA COVID vaccine one day prior to presenting to hospital. Ultrasound (US) with Dopplers of the lower extremities revealed an indeterminate hypoechoic solid appearing avascular 5 cm lesion in the left distal thigh that was inseparable from the distal femoral vein (FV) and popliteal vein (PV). The patient was subsequently admitted and initiated on anticoagulation for her PE.

Two days later, the patient became unresponsive and hemodynamically unstable with blood pressure of 70/30, tachycardia with heart rate 150 beats per minute, and oxygen saturation of 85%, was transferred to the ICU, and was treated with thrombolysis for massive PE. Repeat echocardiogram showed left ventricular (LV) systolic flattening, moderately reduced RV function, and severely increased right ventricular systolic pressure (RVSP), which had been normal on the initial echocardiogram. Repeat US of the lower extremities was unchanged. MRI of the hip confirmed the same complex lesion that was inseparable from the distal FV, and it was ultimately suspected to be a venous aneurysm with a partially thrombolysed internal thrombus.

One week after admission, the patient was discharged on enoxaparin 135 mg subcutaneously every 12 h. In outpatient follow-up at 36 weeks gestation, she had factor Xa levels that were within the therapeutic range (0.4–1.2 u/mL). At nearly 38 weeks gestation, the patient had an uncomplicated spontaneous vaginal delivery and was discharged on therapeutic tinzaparin with a plan for 6 months of therapeutic anticoagulation. Four weeks post-partum she reported left calf pain, but was otherwise doing well.

**Fig. 1:**
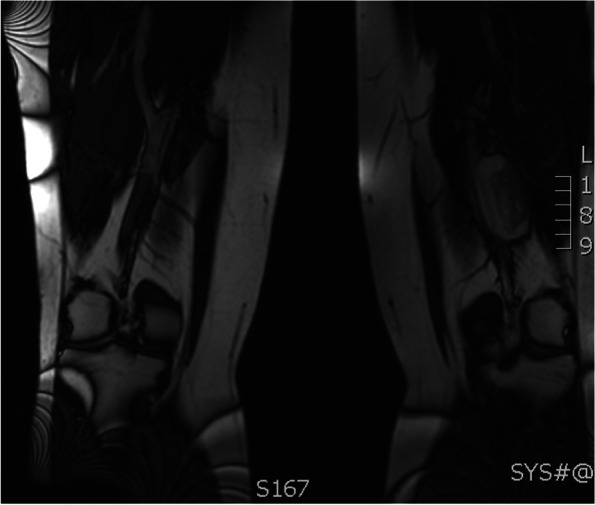
MRI (Coronal view) demonstrating saccular aneurysmal dilation measuring 43 × 31 × 68 mm

**Fig. 2:**
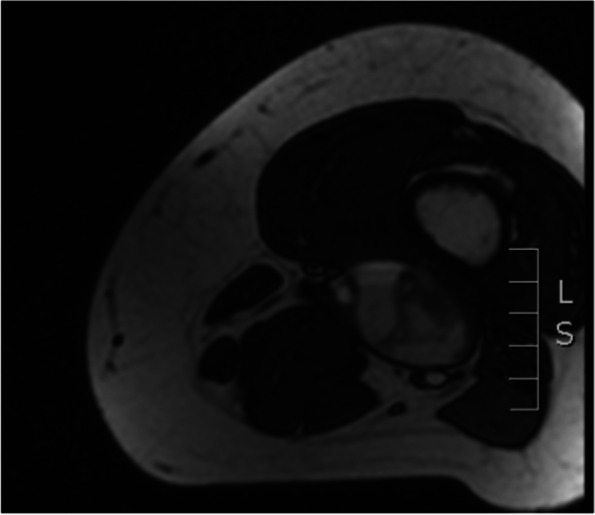
MRI (transverse view) demonstrating thrombus with aneurysm sac, mixed signals consistent with thrombosis of varying ages, and non-occlusive thrombosis within the profunda femoris vein with moderate stenosis

Shortly prior to the completion of 6 months of therapeutic anticoagulation, the patient presented to the ED with dyspnea, palpitations, and presyncope. V/Q scan showed extensive PE but did not differentiate between new and chronic embolism. A computed tomography pulmonary angiogram (CTPA) was performed and confirmed acute PE with evidence of RV strain. Due to recurrent PE on therapeutic tinzaparin, a thrombophilia work-up was completed, which was negative, and tinzaparin dosing was increased to 115% of the weight-based dose. A repeat US with Doppler revealed the known duplicated FV with a large varix and suspicion of a new non-obstructing acute thrombus within the varix. Thrombosis experts were consulted, and the patient was maintained on supratherapeutic tinzaparin dosing and urgently referred to Vascular Surgery for consideration of surgical correction. A repeat MRI demonstrated saccular aneurysmal dilation measuring 43 × 31 × 68 mm (Fig. 1), increased in size from previous, and demonstrated progression of thrombus within the aneurysm sac. There was also non-occlusive thrombus within the profunda femoris vein (Fig. 2).

A pre-operative venogram demonstrated that the patient had a duplicated PV and a hypertrophied profunda vein branch feeding collaterals circumventing the aneurysm to the FV. The options of both aneurysmorrhaphy and venous ligation were discussed in detail with the patient in light of the duplicated PV and collateral circuit. The risk of post thrombotic syndrome with ligation was discussed, but given her life-threatening presentation, the patient favoured complete ligation. Intraoperatively, she was found to have a 6.5 cm aneurysm of the PV, with normal vein segments proximally and distally. The PV was ligated proximally and distally to the aneurysmal segment. Following surgery, the patient was discharged home on tinzaparin. She was transitioned to warfarin instead of a direct oral anticoagulant as she was breastfeeding, and the plan was for post-operative anticoagulation for a total of 6 months. A repeat V/Q scan revealed residual unmatched perfusion defect but no features of chronic thromboembolic pulmonary hypertension (CTEPH). Given the evidence of residual clot burden, the decision was made to extend anticoagulation to one year post-operatively and then consider low-dose Aspirin for secondary prevention of VTE.

## Discussion

PVA are rare with an incidence of 0.2–0.5%, occurring more commonly in women, and they are typically saccular (as opposed to fusiform) [1–3]. Current evidence and guidance is based on case reports due to their rarity. The proposed causes include trauma, vascular hypertension, inflammation, connective tissue disease and/or congenital abnormalities [1]. Many patients with PVA are asymptomatic. Only 20% have been reported to present with palpable masses in the popliteal fossa [7]. The most common presenting symptoms are those associated with PE given the significant VTE risk that PVA pose, as was the case for our patient [2–6]. PVA are extremely important to identify and treat given the potential for fatal outcomes. Diagnosis can be made with several imaging modalities, but duplex US is reliable to accurately identify and characterize PVA size, morphology, intra-aneurysmal thrombus, and is safe to be repeated for reassessment pre- and post-operatively, especially in the case of pregnancy [3].

In our case, the primary cause of PVA was unknown. Pregnancy and the post-partum period are prothrombotic states for a number of reasons and are associated with a four- to fivefold increased risk of VTE compared to non-pregnant women [8]. The physiologic and anatomical changes responsible for this increased risk include increased production of factors V, VII, VIII, IX, X, and XII, von Willebrand factor, fibrinogen, decreased protein S activity, decreased fibrinolysis, and progesterone-mediated veno-dilation, increased blood volume and venous hypertension. Anatomically, compression of the inferior vena cava and pelvic veins as the uterus enlarges contributes to venous dilation, hypertension, and stasis [8, 9]. Increased venodilation and blood volume have been shown to result in an increase in diameter of lower extremity vasculature, including the PV [9]. It could therefore be hypothesized that pregnancy may have played a role in causing and/or contributing to the formation and growth of the PVA, as well as promoting intra-aneurysmal thrombus formation. A similar explanation has been proposed in a patient who presented with pain in the posterior fossa post-partum with a history of a popliteal mass identified in her second trimester who was found to have a dissecting PVA [10]. The recurrence of PE in the post-partum period in our case reinforces this as a high-risk period, which extends up to 12 weeks post-partum [9].

In terms of management, thrombolytic therapy has been recommended in the acute setting for patients with PVA and PE, as it can both improve cardiorespiratory status and reduce or eliminate the aneurysmal thrombus, facilitating operative repair [4]. Inferior vena cava filters placement remains controversial, but safe and successful use has been reported in an effort to reduce the risk of perioperative VTE in a patient with intra-aneurysmal thrombus with extension into the vein [11].

For long-term management, the literature does not currently support anticoagulation alone given the significant risk of recurrent PE, as observed in our case. Rates of recurrent thrombosis while on anticoagulation have been reported as high as 43–80%, highlighting the importance of surgical correction as definitive management [2, 4, 5, 12]. Surgery is recommended for definitive management of PVA as it has been demonstrated to be safe and significantly reduces the risk of recurrent VTE. Operative management was previously recommended regardless of symptoms and/or size, based on the ill-defined but significant risk of PE [4, 5, 7, 13]. In the most recent review of management of PVA, the specific recommendation for surgery is refined to include patients with large PVA (defined as > 20 mm) with turbulent flow, which was found to be associated with higher risk of thrombus formation [1]. Surgical options include aneurysmectomy with lateral venorrhaphy, resection and end-to-end anastamosis, resection and autologous or alloplastic graft interposition, and ligation of the proximal and distal vein [3, 14]. Aneurysmectomy with lateral venorrhaphy to remove the source of embolism and maintain venous flow is the preferred approach based on technical feasibility and surgical success rates [3]. Our approach differed from this based on our patient’s unique anatomy, recurrent presentations and patient preference. Ligation as a means of removing the source of embolism was favoured. In a recent review, only 3% of patients who underwent surgical intervention for PVA had a recurrent aneurysm, and this was specifically following patch plasty. There have not been any reports of recurrent PVA following aneurysm resection [1].

In our case, the patient’s pregnancy added a layer of complexity given the persistent risk of thrombosis with an uncorrected PVA and pregnancy, high risk of possible recurrence with interruption of anticoagulation for surgery, and general risks of undergoing surgery while pregnant. There are few reports of PVA in pregnancy, which further complicated decision-making. In one report of a patient with PVA diagnosed in the setting of PE at 8 weeks gestation in a pregnancy achieved by in vitro fertilization, medical management with LMWH was pursued for the duration of pregnancy based on patient preference [14]. Following delivery, she was maintained on warfarin while breastfeeding, and underwent surgical aneurysmectomy 7 months post-partum [14]. In another case of external iliac venous aneurysm in pregnancy, the patient elected to terminate her pregnancy and undergo surgical correction [15]. Lastly, a case of dissecting PVA has been reported in the post-partum period, and this patient successfully underwent excision and ligation [10].

The duration of anticoagulation post-operatively has varied from 3 weeks to 6 months of therapy based on the literature [2, 4]. Pneumatic compression devices are suggested as a non-pharmacological strategy post-operatively, and have been previously prescribed for 3 months post-operatively [1, 2]. In a previous report of PVA in a pregnant patient who underwent aneurysmectomy post-partum, anti-coagulation was continued for at least 6 months [14]. Our patient elected to continue anticoagulation beyond 6 months post-operatively given the residual PE burden seen and strong aversion to recurrence given the severity of her initial presentation.

## Conclusion

PVA are a rare but important clinical entity to consider in patients with VTE and pose a significant risk of recurrent VTE if left untreated [6]. The risk of VTE is further increased in the setting of pregnancy and the post-partum period, as demonstrated in this case of PVA with PE and recurrent PE despite therapeutic anticoagulation in the post-partum period. This case illustrated the importance of screening for PVA as a source of VTE, the acute and chronic management of PVA in the setting of pregnancy, and the high vigilance with which these patients need to be followed while on anticoagulation alone given the significant risk of recurrent VTE, compounded by the prothrombotic states of pregnancy and post-partum. Reassessment with frequent follow-up and serial US Dopplers in this period while awaiting surgery may be beneficial to facilitate changes in dosage if new or increased size of intra-aneurysmal thrombus is identified.

The recommended surgical management of PVA with PE with surgical resection of the aneurysm is complicated in the setting of pregnancy. When surgery can be safely performed, as in this case, it should be pursued to reduce the risk of recurrent VTE and long-term complications, for example CTEPH. The ideal duration of post-operative anticoagulation remains unclear, and should likely be decided on based on risks, benefit, values, and shared decision making with the patient and their care provider.

## Data Availability

Not applicable.

## Abbreviations

CTEPH: Chronic thromboembolic pulmonary hypertension
CTPA: Computed tomography pulmonary angiogram
ED: Emergency department
ICU: Intensive care unit
LV: Left ventricular
PV: Popliteal vein
PVA: Popliteal vein aneuryms
PE: Pulmonary embolism
RV: Right ventricular
RVSP: Right ventricular systolic pressure
US: Ultrasound
VTE: Venous thromboembolic events
V/Q scan: Ventilation/Perfusion Scan
FV: Femoral vein
